# Determinants of Innate Immunity in Visceral Leishmaniasis and Their Implication in Vaccine Development

**DOI:** 10.3389/fimmu.2021.748325

**Published:** 2021-10-12

**Authors:** Greta Volpedo, Thalia Pacheco-Fernandez, Parna Bhattacharya, Timur Oljuskin, Ranadhir Dey, Sreenivas Gannavaram, Abhay R. Satoskar, Hira L. Nakhasi

**Affiliations:** ^1^ Departments of Pathology and Microbiology, Wexner Medical Center, The Ohio State University, Columbus, OH, United States; ^2^ Laboratory of Emerging Pathogens, Division of Emerging and Transfusion Transmitted Diseases, Center for Biologics Evaluation and Research, Food and Drug Administration, Silver Spring, MD, United States

**Keywords:** innate immunity, vaccine, metabolic regulation, visceral leishmaniasis, live attenuated leishmania vaccines, trained immunity, metabolomics, immune-regulation

## Abstract

Leishmaniasis is endemic to the tropical and subtropical regions of the world and is transmitted by the bite of an infected sand fly. The multifaceted interactions between *Leishmania*, the host innate immune cells, and the adaptive immunity determine the severity of pathogenesis and disease development. *Leishmania* parasites establish a chronic infection by subversion and attenuation of the microbicidal functions of phagocytic innate immune cells such as neutrophils, macrophages and dendritic cells (DCs). Other innate cells such as inflammatory monocytes, mast cells and NK cells, also contribute to resistance and/or susceptibility to *Leishmania* infection. In addition to the cytokine/chemokine signals from the innate immune cells, recent studies identified the subtle shifts in the metabolic pathways of the innate cells that activate distinct immune signal cascades. The nexus between metabolic pathways, epigenetic reprogramming and the immune signaling cascades that drive the divergent innate immune responses, remains to be fully understood in *Leishmania* pathogenesis. Further, development of safe and efficacious vaccines against Leishmaniasis requires a broader understanding of the early interactions between the parasites and innate immune cells. In this review we focus on the current understanding of the specific role of innate immune cells, the metabolomic and epigenetic reprogramming and immune regulation that occurs during visceral leishmaniasis, and the strategies used by the parasite to evade and modulate host immunity. We highlight how such pathways could be exploited in the development of safe and efficacious *Leishmania* vaccines.

## Introduction

Protozoan parasites of the genus *Leishmania* are the causative agents of leishmaniasis, a spectrum of vector-borne neglected diseases affecting over 12 million people worldwide with growing geographical extension (1, 2). The clinical manifestations of leishmaniasis differ widely, depending principally on the causative species. An estimated 20 different *Leishmania* spp. cause the three main clinical disease manifestations: cutaneous, mucocutaneous, and visceral leishmaniasis (3). Among these, the visceral form is fatal if not treated. Most cutaneous leishmaniasis (CL) and visceral leishmaniasis (VL) patients develop long-term protective immunity after cure from the infection, indicating the feasibility of developing an effective prophylactic strategy against leishmaniasis (4, 5). However, no vaccine is currently available against human leishmaniasis.

*Leishmania* is a digenetic parasite, whose life cycle includes two hosts: the insect vector, and a vertebrate host. Following the bite of an infected sand fly, the obligate intracellular parasite *Leishmania* rapidly infects its host cells (2, 6). A better understanding of the spectrum of immune responses following infection with *Leishmania* parasites is required to develop more effective preventative approaches, as the host immune response is a key determinant of the outcome of leishmaniasis (7). Following inoculation, innate cells such as neutrophils, inflammatory monocytes, macrophages, dendritic cells (DCs), mast cells, and natural killer (NK) cells create either a permissive or hostile environment for the parasite through their effector functions, and subsequently modulate the adaptive immune responses towards host susceptibility or resistance. Although the adaptive branch of the immune response is crucial to control leishmaniasis, it has been extensively reviewed elsewhere and it is out of the scope of this review. Specifically, we focus on the innate immune responses in visceral leishmaniasis due to its significant mortality (7, 8).

*Leishmania* parasites have evolved highly successful strategies to evade the microbicidal activity of neutrophils, to prime infected macrophages towards an anti-inflammatory/alternative phenotype, and to undermine the Th1 polarizing functions of DCs, thereby attenuating the host protective adaptive immune responses (9–11). These altered immunological characteristics of the host cells upon infection are often induced by changes in the host metabolic pathways. Thus, there has been an emerging interest in profiling host metabolomics in the context of immune regulation, and especially to understand how metabolites and metabolic processes such as oxidative metabolism, glycolysis, and glutaminolysis can influence immune cell proliferation, differentiation and effector functions (12, 13). For instance, in pathogen-associated molecular pattern (PAMP)-activated neutrophils, mast cells, and DCs, glycolysis acts as a metabolic effector response that fuels reactive oxygen species (ROS) production, degranulation, and antigen presentation, respectively (13). Furthermore, changes in the metabolomic profile of infected immune cells can alter nutrient availability to the parasite, rendering the intracellular environment either permissive or hostile thus determining the anti-leishmanial activities (14). *Leishmania* parasites can also alter the metabolic pathways of the host cell to their advantage, for example by influencing nutrient uptake (15). It is hypothesized these metabolomic alterations can protect the parasite from elevated temperatures, low pH, and ROS in the parasitophorous vacuole (PV), leading to enhanced survival inside the host cell (15).

Increasing evidence points to a crucial role of innate immune cells in determining the outcome of the infection. This review particularly attempts to highlight the role of the innate immune cells, the metabolomic and epigenetic reprogramming, and immune regulation during VL, and discuss their implications with respect to the development of a vaccine against VL.

## Innate Cells and Their Role in VL

### Neutrophils

#### Role of Neutrophils in VL Pathogenesis

**Figure 1:**
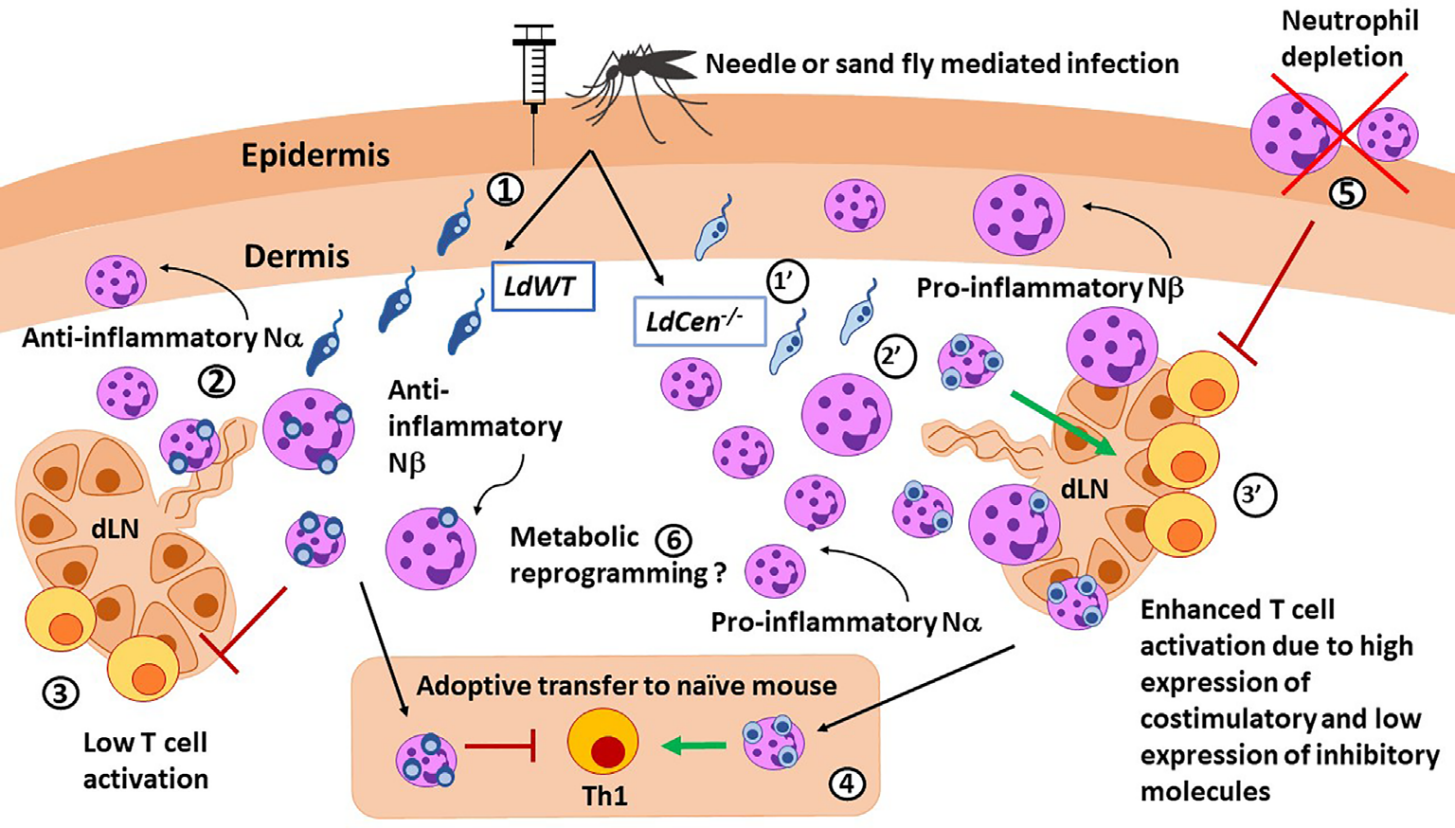
Role of neutrophils in immunity against *Leishmania* parasites. *LdWT* (1) or *Ldcen^-/-^* (1’) parasites are injected with a needle or by the sand fly vector. Compared with *LdWT* infection (2), *LdCen^−/−^* (2’) parasites induce higher recruitment of neutrophils, especially the smaller Nα subtype, to the ear dermis and ear draining lymph nodes (dLN), which were predominantly proinflammatory in nature. Similar Nα and Nβ subtypes were reported in live attenuated HSV vaccine studies. Neutrophils from ear dLN of *LdCen^−/−^*-immunized (3’) mice exhibited heightened expression of costimulatory molecules and attenuated expression of coinhibitory molecules necessary for higher T cell activation compared to *LdWT* (3). 4) Adoptive transfer of neutrophils bearing *LdCen^−/−^* parasites induced an increased Th1 response in naive mice compared to *LdWT*. 5) Neutrophil depletion significantly abrogated Ag-specific CD4+ T cell proliferation in *LdCen^−/−^*-immunized mice and impaired protection against virulent challenge. Conversely, replenishing of neutrophils significantly restored the *LdCen^−/−^*-induced host-protective response. 6) Neutrophils can undergo metabolic reprogramming to acquire trained immunity characteristics as shown in BCG vaccination studies.

Neutrophils are the first innate immune cells recruited to the dermal site of *Leishmania* infection in response to several factors derived either from the host, the sand fly, or the parasite itself (16, 17) (**Figure 1**). Neutrophils’ anti-parasitic activities include phagocytosis, formation of neutrophil extracellular traps (NETs), as well as the release of reactive species (RNS and ROS) and granule-derived toxic compounds in the local environment or into the phagosome (18–23) (**Figure 1**). Additionally, neutrophils actively participate in modulating the adaptive immune responses. Specifically, several cytokines and granule proteins secreted by neutrophils such as interleukin (IL)-12, interferon (IFN)-γ, tumor necrosis factor (TNF)-α, and neutrophil elastase can induce T cell activation (24–27). Alternatively, IL-10, transforming growth factor (TGF)-β (**Figure 1**), and eicosanoids (thromboxane A2) from neutrophils suppress T cell activity (24–26, 28).

The divergent immune responses observed in neutrophils may be induced by changes in the metabolism of *Leishmania* infected cells (27). For instance, *L. donovani*-infected neutrophils undergo a rapid upregulation of glycolytic enzymes at 6 hours post infection. Inhibition of glycolysis prior to infection led to significantly higher parasitic burdens in neutrophils, by inhibiting the generation of ROS production due to reduced NADPH oxidase activity (13, 27). Metabolic reprogramming in neutrophils may affect other functions in addition to microbicidal activities and remains an area of active investigations in *Leishmania* and other pathogenic agents.

Neutrophils have been shown to play a protective role during *L. donovani* infection (11). The rapid recruitment of neutrophils during *L. donovani* infection promotes resistance *via* the induction of an IFN-γ–dominant Th1 response in mice (29), and canine models of VL (30). Different visceralizing species of *Leishmania* can differentially affect neutrophil activities. For example, *L. donovani* infection can mediate recruitment, survival, and deactivation of neutrophils *via* the lipoxin A4 receptor (16, 31). Conversely, *L. infantum* interaction with the lipoxin A4 receptor promoted the activation of neutrophils characterized by an increase in NET formation (32). These divergent neutrophil roles due to different *Leishmania* species were identified by manipulating the neutrophils in animal models by treatment with neutrophil depleting antibodies that may have certain limitations (17, 18, 31). In addition to metabolite receptors such as lipoxin A4, vector saliva derived factors such as sialogenins and yellow salivary protein may also contribute to the diverse neutrophil-specific responses including FasL-mediated apoptosis in different experimental models (33, 34). Due to the important role played by neutrophils in early immunity, these populations have been studied in human VL as summarized in the following sections.

#### Role of Neutrophil Interactions With Other Immune Cells in Leishmania Immune Response

Neutrophils, in addition to their independent role in orchestrating the early cytokine/chemokine responses, also interact with other immune cell types including DCs, macrophages, NK cells, B cells, and T cells (35). Since macrophages are the principal host cells for *Leishmania* parasites, the crosstalk between neutrophils and infected macrophages has been studied in VL. The interaction between neutrophils and other immune cells has been shown to affect *Leishmania* infection and innate immune functions upon acquiring parasitized neutrophils (36–40). The species of *Leishmania* parasites, the host genetic background, and the apoptotic or necrotic nature of the neutrophils determine parasite transfer from neutrophils to macrophages, and the subsequent outcome of their interaction with macrophages (23, 37). The crosstalk between neutrophils and macrophages/DCs in VL remains to be studied (41), although it is known that when human macrophages are co-cultured with neutrophils previously infected with *L. infantum* in the presence of salivary gland sonicate (SGS), they acquired significantly more parasites from neutrophils. These macrophages also produced more TGF‐β and prostaglandin E2 (PGE2), suggesting the importance of neutrophil-macrophage crosstalk (42). Parasitized neutrophils may facilitate the uptake by DCs *via* the expression of apoptotic markers that lead to decreased DC activation and impaired cross-presentation for CD8+ T cell activation. Although CD11b on neutrophils may interact with DC‐SIGN of DCs and induce secretion of TNF‐α, such interaction remains to be demonstrated in VL (41). A predominant immunoregulatory role for neutrophils in regulating DC functions and subsequent T cell differentiation *via* DCs during *Leishmania* infection has also been reported (16, 41). The role of alarmins released during degranulation of neutrophils and their role in altering DC functions remains to be studied in VL (43).

#### Phenotypic Heterogeneity of Neutrophils

Heterogenous subsets of neutrophils identified based on size and granularity have been observed to perform either pathogenic or protective roles in autoimmune diseases, tumors, viral diseases and vaccines (44–46). Accordingly, Nα and Nβ neutrophil sub-populations have been described previously in the context of murine vaccination with attenuated New York vaccinia virus (NYVAC-C3). In this paper, Pilato, et al. reported higher expression of activation markers (CD11c, CD80, and CD86) in Nβ neutrophils. Compared to Nα cells, Nβ had greater capacity to induce antigen-specific CD8^+^ T-cell activation against the virus (46). Similar studies elucidating the functional roles of distinct neutrophil subsets revealed diverse roles of neutrophils in VL. Characterization of recruited dermal neutrophils in C57BL/6 mice during VL revealed the presence of heterogeneous Nα and Nβ neutrophil populations; Nα being the predominant population susceptible to infection (47). Further, studies in human VL identified the presence of a subset of HLA-DR^+^ low density circulating neutrophils that expressed elevated levels of arginase-1 and IL-10. These neutrophils failed to stimulate autologous T cell proliferation and promoted T cell exhaustion due to higher PD-L1 on their surface and elevated PD-1 expression by lymphocytes (48). The divergent functional specialization of neutrophil subsets observed in VL suggests that their role in other *Leishmania* infections warrants further studies.

#### Neutrophils and Their Role in Vaccine Induced Immunity

In addition to the innate immune functions either independently or through crosstalk between macrophages and DCs illustrated in the previous section in the context of VL, neutrophils have been shown to directly present antigens and promote T cell activation (49–51). Due to their capacity to perform antigen presentation to naïve T cells, similarly to other APCs, neutrophils may be particularly relevant in vaccine immunity. Indeed, a neutrophil-mediated T cell priming response was illustrated in numerous vaccines, including modified vaccinia Ankara virus, poxvirus, and live attenuated tuberculosis vaccine (46, 52, 53). The protective role of neutrophils following immunization against tuberculosis is demonstrated when depletion of neutrophils abrogated the induction of Th1-specific responses (52). Similarly, neutrophil depletion significantly abrogated Ag-specific CD4^+^ T cell proliferation in live attenuated *centrin* gene-deleted *L. donovani* (*LdCen^-/-^*)-immunized mice and impaired protective immunity against virulent *L. donovani* challenge (47). In contrast, depletion of neutrophils in animals immunized with killed *L. major* + CpG vaccine enhanced protection upon sandfly mediated challenge (19). In *LdCen* ^-/-^ immunization studies, distinct functional roles of neutrophil subsets were observed. Specifically, Nα subsets from *LdCen*−/− infected mice expressed significantly higher levels of costimulatory molecules along with attenuation of coinhibitory molecules, resulting in significantly higher CD4^+^ T cell activation compared with the Nα population from *L. donovani* wild type-infected mice (47). More studies are needed to elucidate the role of neutrophil subsets in other anti-leishmanial vaccination regimens.

In addition to the role of neutrophils in adaptive immune responses, recent vaccination studies have shown that neutrophils may also acquire non-specific memory due to epigenetic and metabolic changes. This process termed “trained immunity” is being intensely studied in neutrophils and other cell types. Metabolic reprogramming induced by immunological signals determines the epigenetic changes that orchestrate trained immunity (54). These epigenetic modifications are usually broad, not pathogen-specific, and include chromatin changes to a gene or locus (55). A recent report form Moorlag, et al. demonstrated that neutrophils acquire trained immunity after Bacillus Calmette-Guérin (BCG) vaccination. Furthermore, trained neutrophils showed long-term reprogramming and maintained their enhanced activation up to 3 months after vaccination (56). Similar data is not available in leishmaniasis models. The broader role of neutrophils in vaccine immunity in general and their capacity to acquire trained immunity characteristics in particular, are of significant interest although durability and thus relevance of trained immunity in neutrophils remain to be demonstrated in anti-*Leishmania* vaccines.

## Monocytes

**Figure 2:**
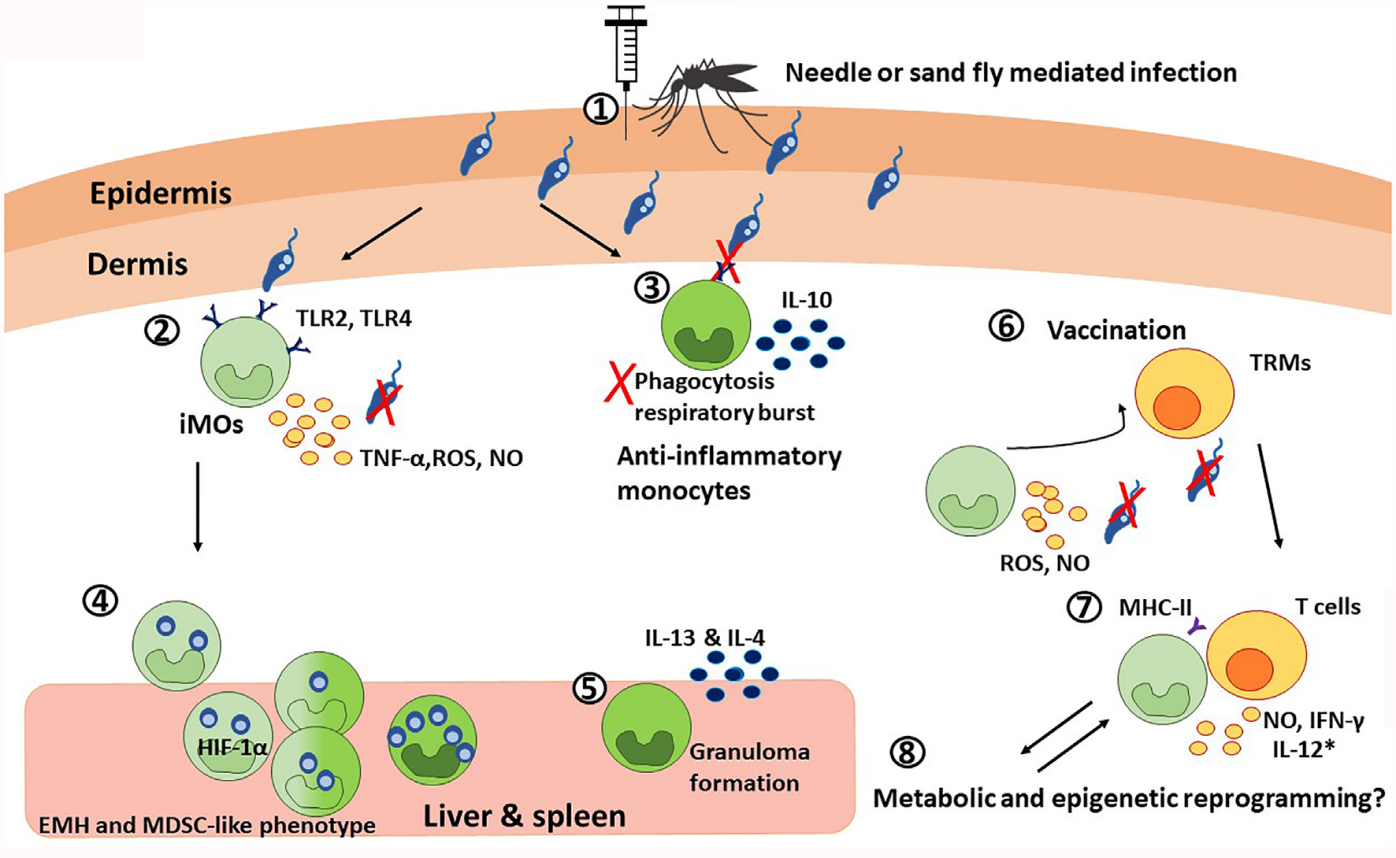
The paradoxical roles of monocytes during visceral leishmaniasis. 1) Monocyte chemotaxis to the infection site is mediated in a CCR2-dependent manner. During the blood meal, this process is additionally potentiated by sand fly salivary components. 2) The inflammatory monocytes, iMOs (light green), recognize *Leishmania* trough TLR2 and TLR4. TLR recognition, together with the IFN-γ released by neutrophils, induce the secretion of TNF-α, ROS, and NO that results in parasite killing. Although the specific cytokine regulation is species-specific. 3) Particularly during VL, *Leishmania* can downregulate TLR2 and TLR4 signaling, dampening phagocytosis and respiratory burst, and promoting IL-10 production. This way, the parasite promotes its own survival by favoring the anti-inflammatory monocyte profile (dark green). 4) Infected iMOs migrate rapidly to the liver and spleen, where they undergo extramedullary hematopoiesis (EMH) and acquire a HIF-1α-mediated MDSC-like phenotype, that is permissive for parasite survival, as observed in VL patients. 5) In the liver and spleen, anti-inflammatory monocytes are necessary for granuloma formation *via* the production of IL-13 and IL-4, which has a protective role during VL. 6) In the context of vaccination, iMOs and T cells interplay is critical for memory responses. On the one hand, skin resident CD4+ memory T cells (TRMs) recruit iMOs to the infection site, where they produce ROS and NO to control the infection. 7) At the same time, iMOs activate T cell responses by presenting antigens *via* MHC-II and producing NO and IFN-γ; and with the use of co-adjuvants targeting monocytic response, iMOs can also produce IL-12 (*). 8) Finally, it is possible that monocytes undergo metabolic and epigenetic reprograming during VL.

Monocytes, along with neutrophils, are the earliest immune cell populations to be recruited after infection of *Leishmania* (57) (**Figure 2**). Recruitment of monocytes is modulated by pro-inflammatory cytokines and chemokines after infection with different *Leishmania* species. Monocyte subpopulations exist within a phenotypical spectrum ranging from long-lived patrolling monocytes (non-classical) that mainly monitor the vasculature as part of normal physiology (58) to short-lived inflammatory (classical) monocytes (iMOs, CD11b+ CX3CR1^lo^ Ly6C^hi^ CCR2+ CD115+) (59, 60). During the bloodmeal, *Leishmania* is injected along with sand fly salivary components, which modulate inflammatory responses that lead to CCR2-mediated chemotaxis of iMOs to the infection site (61), reviewed in (62, 63). iMOs play contradictory roles in different infections: while they mediate host defense against toxoplasmosis (64) and malaria (65), a detrimental role in VL was demonstrated (51). Monocytes can recognize *Leishmania* parasites *via* Toll-like receptors (TLR)s and produce pro- and anti-inflammatory cytokines that can play paradoxical roles during leishmaniasis (66–69) (**Figure 2**). The expression of TLRs and the molecules on the parasite surface could also generate a diverse range of monocytic responses during infection with different *Leishmania* species. For instance, *L. donovani* infection of monocytes inhibits oxidative burst and antigen presentation and activates IL-10 expression *via* downregulation of TLR2 and TLR4 signaling (70, 71). In contrast, during *L. tropica* infection, monocytes showed higher expression of TLR9 in addition to TLR2/TLR4, and elevated TNF-α that resulted in a decrease of inducible nitric oxide synthase (72). Furthermore, *L. braziliensis* infection of human monocytes showed enhanced production of TNF-α and higher TNF-α/IL-10 ratios, and controlled parasite burdens more effectively compared to *L. infantum* infection (66). In addition to their role in pathogenesis, monocytes play an important role in granuloma formation, necessary for parasite clearance in murine models of *L. donovani* infection. However, an IL-17^-/-^ mouse model of *L. donovani* infection showed diminished monocyte and neutrophil infiltration resulting in smaller isolated granulomas in the liver and spleen, yet controlled parasite burden due to elevated IFN-γ production (73).

Studies of active VL patients in Brazil and India showed an increase of CD14^+^ monocytes, and elevated expression of IL-10, which affects macrophage polarization and the outcome of the disease (74, 75). Other studies with Indian VL patients reported an anti-inflammatory (non-classical) monocyte response in the peripheral blood characterized by reduced expression of TLR2 and TLR4, chemokine receptors and adhesion molecules, as well as impaired phagocytosis and oxidative burst, important for the elimination of *Leishmania* parasites (70, 76). These observations illustrate the critical role of monocytes in the early modulation of host immune responses and the clinical outcome of *L. donovani* infection. The divergent engagement of TLRs and associated immune responses following infection of monocytes with different *Leishmania* species suggests that targeting these interactions could be exploited in the development of effective anti-parasitic strategies.

### Role of Monocyte Subsets in the Pathogenesis of VL

A growing body of research is focused on elucidating the role of sub-populations of monocytes such as iMOs, intermediate, and non-classical patrolling monocytes in *Leishmania* infection. Monocytic subsets (CD14+ CD16- and CD14+ CD16+) of human origin displayed significantly increased phagocytic capacity and intracellular NO production when infected with *L. infantum*, compared to *L. braziliensis* (77). iMOs contribute to parasite control at the lesion site in CL (78), but they play a detrimental role in VL (59). Similarly, different subsets of resistant (Ly6C^lo^/M1-like) or permissive (Ly6C^hi^/M2-like) monocytes have also been reported in VL (79). Our previous studies demonstrated that iMOs (CD11b+ Ly6C^hi^) are rapidly recruited into the spleen and liver within 24 hours of *L. donovani* infection in a CCR2-dependent manner, such that iMOs can make up to 15-25% of the myeloid cells in these organs (**Figure 2**) (59). The iMOs recruited to the spleen during *L. donovani* infection can acquire a hypoxia‐inducible factor (HIF)-1α mediated myeloid-derived suppressor cells (MDSC) like phenotype, which promotes a chronic infection (80). *L. donovani* infection of hamsters has been shown to induce extramedullary hematopoiesis of myeloid cells in the spleen resulting in the accumulation of monocytes that support parasite replication (81). Migration of Ly6C^hi^ iMOs to the spleen and liver is facilitated by STAT-1 signaling (59, 82). Therefore, altering monocyte migration to spleen and liver by immunomodulatory agents could be a potent parasite control strategy (83). Indeed, therapeutic reduction of the iMOs influx to the visceral organs with Ibrutinib, is shown to reduce susceptibility to *L. donovani* infection in mice (84).

### Role of iMOs in Vaccine Induced Immunity

Due to the pluripotent nature of monocytes to differentiate into macrophages and DCs, it is important to understand the early interaction between monocytes and *Leishmania* parasites. A recent study showed that iMOs are necessary to control *L. major* infection during challenge in a healed C57Bl/6 mouse model (85). iMOs are the main inducible nitric oxide synthase (iNOS) producers during secondary infections (86). Specifically, skin resident CD4+ memory T cells (TRMs) responsible for controlling the secondary infection recruit iMOs to the challenge site. These recruited iMOs reduced the parasite burden even in absence of CD44+ IFN-γ+ effector T cells by producing ROS and nitric oxide (NO) (85). iMOs also play a critical role in initiating differentiation of T cells to acquire a memory phenotype by secreting IL-18 and IL-15 in *Listeria monocytogenes* models, suggesting that iMOs could be an analogous source of these cytokines in *Leishmania* infections, though this needs to be formally demonstrated (87). Thus, due to their roles in parasite clearance at the challenge site and potentially in initiating a TRM response, targeting iMOs could improve efficacy of anti-*Leishmania* vaccines. For example, CpG oligonucleotides, when used as adjuvants, have been shown to induce IL-12 in monocytes (88). Studies of Leishmune*®*, a vaccine for canine VL, have elucidated the critical role of monocytes in relation to vaccine efficacy. Monocytes and neutrophils from vaccinated dogs showed increased phagocytic activity, NO and IFN-γ production, expression of TLRs, costimulatory molecules, and MHC-II (89, 90). The importance of monocytes relies not only on their early immune responses, but also in their capacity to activate an effective adaptive response, which is crucial in any vaccination design.

As described in the neutrophil section, there is an emerging interest in exploring the role of epigenetic and metabolic reprogramming in the context of trained immunity in monocytes (**Figure 2**). For example, Bacillus Calmette-Guérin (BCG) and β-glucan from *Candida albicans* can induce epigenetic changes in histone trimethylation at H3K4 in monocytes and macrophages to promote trained immunity (91). In particular, it seems that aerobic glycolysis induced by the activation of Akt, mammalian target of rapamycin complex 1 (mTORC1), and HIF-1 is responsible for cellular activation and trained immunity to BCG vaccination (92). Similarly, β-glucan from *Candida albicans* is shown to induce non-specific trained immunity in monocytes that conferred protection against *L. braziliensis*, by promoting IL-1β signaling and inducing epigenetic changes that result in upregulation of IL-32 (93). However similar trained immunity has not been demonstrated in other *Leishmania* species. These studies highlight how epigenetic and metabolic reprogramming can have profound effects on pathogen immunity. However, little is known about how such mechanisms are operating during *Leishmania* infections overall and how they can be exploited for the development of a pan-*Leishmania* vaccine including protection against visceral leishmaniasis. Since the life span of innate immune cells is limited, trained immunity has been shown to last up only up to one year (94). It is possible that in *Leishmania* endemic areas, where individuals are constantly exposed to *Leishmania* parasites, trained immunity can be repeatedly induced and could play a role in durable protection. Therefore, similar to the BCG vaccine, it may be desirable for efficacious vaccines against VL to target the induction of a robust trained immunity in addition to strong adaptive immune responses that are likely long-lasting.

## Macrophages

**Figure 3:**
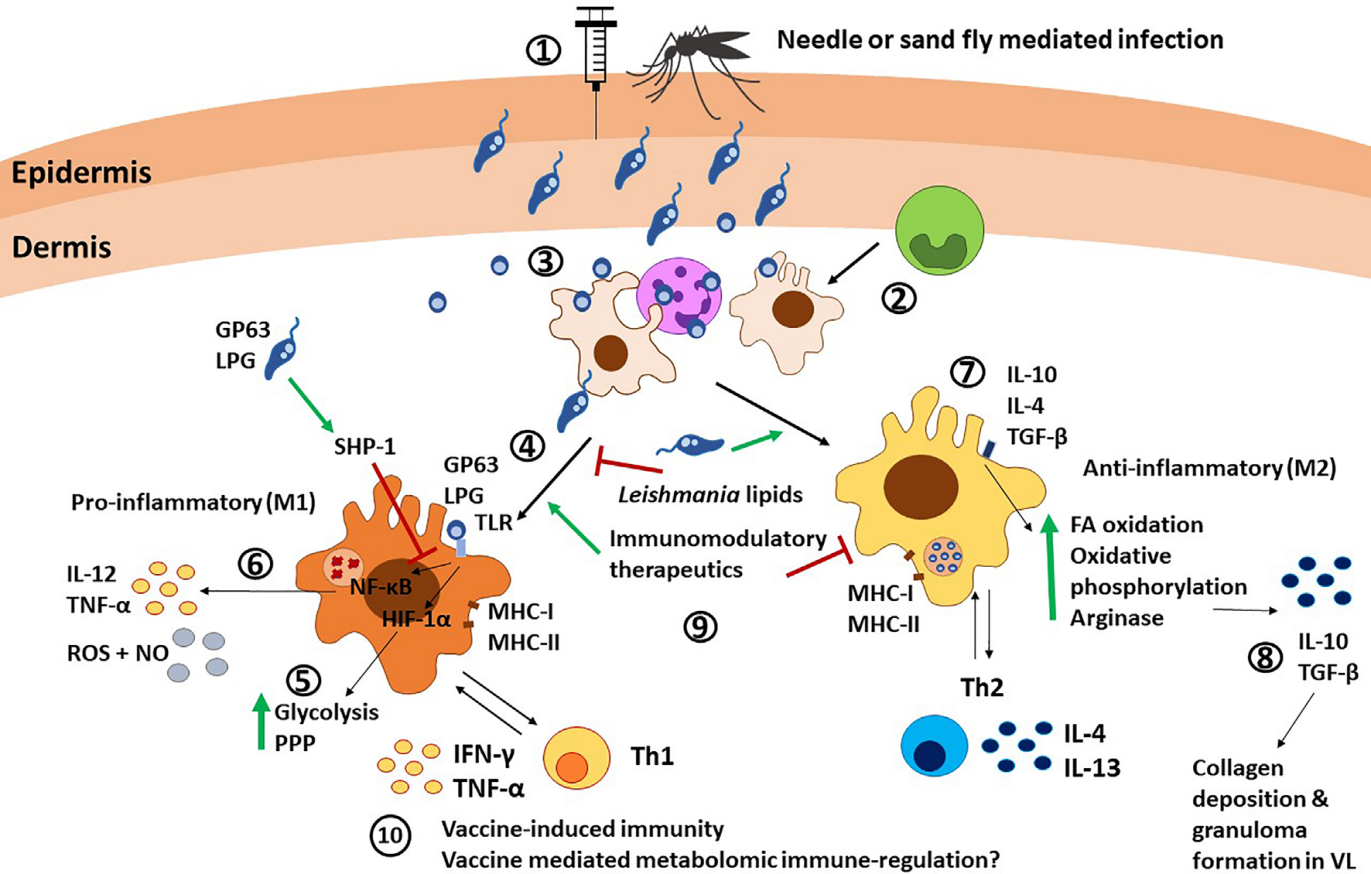
Macrophage immunity against *Leishmania* parasites. 1) *Leishmania* promastigotes are injected into the host *via* needle injection or sand fly injection. 2) Monocytes differentiate into macrophages at the infection site. 3) Macrophages are recruited by neutrophils and infected after neutrophils release *Leishmania* parasites, or as a consequence of phagocytosis of necrotic neutrophils. 4) Macrophages are the canonical host for *Leishmania* and can polarize towards M1 after TLR stimulation *via Leishmania* molecules such as LPG and GP63. 5) TLR signaling induces HIF-1α and subsequent upregulation of glycolysis and the pentose phosphate pathway (PPP). 6) TLR signaling also leads to NF-κB translocation to the nucleus and subsequent release of IL-12, TNF-α, and NO. SHP-1 upregulation by the parasite GP63 and LPS can interfere with this pathway. 7) On the other hand, stimuli such as IL-10, IL-4, and TGF-β can polarize macrophages towards an M2 anti-inflammatory phenotype, characterized by upregulation of fatty acid (FA) oxidation, oxidative phosphorylation and arginase production. 8) M2 macrophages produce IL-10 and TGF-β, which mediate collagen deposition and granuloma formation in VL. 9) Different factors such as *Leishmania*-derived lipids, immunomodulatory therapeutics and Th cells can induce and alter macrophage polarization. 10) The interplay between macrophages and Th1 cells *via* antigen presentation and cytokine release is involved in vaccine-induced immunity. Furthermore, metabolomic reprogramming could influence vaccine immunity, as shown in BCG vaccine, although this remains underexplored in *Leishmania*. PPP, pentose phosphate pathways; FA, fatty acid; NO, nitric oxide.

Macrophages are considered the canonical hosts for *Leishmania* in the later stages of infection, following phagocytosis of *Leishmania*-infected apoptotic neutrophils (**Figure 3**) (95). Neutrophils provide the chemical cues (MCP-1/CCL2, MIP-1α/CCL3, MIP-1β/CCL4 and MIP-2/CXCL2) that help recruit macrophages to the infection site (96). In addition, salivary components induce a significant influx of macrophages in a CCL2/MCP-1 dependent mechanism in *L. chagasi* infection (97). Macrophage response to infection is dependent on the interplay of a) the surface molecules on the *Leishmania* parasites, such as glycoprotein-63 (gp63), lipophosphoglycan (LPG) and glycosylphosphatidylinositol (GPI); and b) the receptors which recognize them, such as Toll-like receptors (TLRs) (98) fibronectin, mannose, complement and Fc receptors (**Figure 3**) (99). Binding of *Leishmania* to complement and Fc receptors slows down phagosome maturation (100) and induces IL-10 production (99, 101, 102). TLRs recognize *Leishmania* surface gp63 and activate the NF-κB pathway in macrophages (**Figure 3**) (103). Interestingly, *Leishmania* uses gp63 to negatively regulate ROS production (104) and is shown to be critical for *Leishmania*-mediated inhibition of NLRP3 inflammasome and attenuating IL-1b secretion (105, 106). Macrophages play a dual role in the infection, as they not only serve as permissive hosts, but are also as anti-*Leishmania* effector cells (107). These contrasting roles depend on the microenvironmental signals in the host tissue (108) in response to different *Leishmania* species and on the differential recognition of the parasites. Depending on the stimulus, infected macrophages can polarize towards a functional phenotype within the spectrum ranging from inflammatory macrophages (also called classically activated or M1) and anti-inflammatory macrophages (also called alternatively activated of M2, **Figure 3**). Macrophage polarization can determine the outcome of the disease (37, 109–111). As discussed in the previous section, iMOs usually mature into M1 macrophages, while anti-inflammatory monocytes are more likely to differentiate into M2 macrophages (59, 112). Some of the parasite factors, such as *L. infantum*-derived lipid mediators, can also induce M2 phenotype by preventing IFN-γ-mediated M1 polarization (113). Functionally, macrophages classified within the M1 and M2 spectrum show distinct activities. The relationship between the macrophage phenotype and the immune milieu during *Leishmania* infection is discussed in more detail elsewhere (107). It is important to note that the phenotypic spectrum of macrophages is quite dynamic, and this plasticity confers a wide range of distinct phenotypic characteristics. However, for the purpose of this review we refer to the pro-inflammatory and anti-inflammatory ends of the spectrum as M1 and M2, respectively, as has been previously established in *Leishmania* immunology.

### Types of Macrophages and Their Role in VL

Macrophages of M1/M2 phenotype are identified primarily based on their immunological characteristics. Recent studies have brought to focus distinct metabolic reprogramming that underlies the functional specialization of macrophages in addition to the vector/parasite derived factors that affect the macrophages. *Leishmania* parasite infection of macrophages is accompanied by metabolic changes that help the parasite adapt to the new environment. For example, *L. infantum* parasites optimize their metabolism to compensate for limited resources within macrophages by redistributing host metabolites towards the synthesis of biomass (114). Although the intracellular metabolism of *Leishmania* parasites is widely conserved, significant differences between *L. infantum* and *L. mexicana* infections have been reported (114–116). Tight junctions between the parasites and the parasitophorous vacuole (PV) allow for the bi-directional transport of lipids (14). Because of this, lipid bodies can accumulate in and around PVs and provide amastigotes with a carbon source of polyunsaturated fatty acids (PUFA) (14). Parasite metabolites themselves can affect the polarization of the macrophages. For instance, the infective metacyclic stages of *L. infantum* showed increased levels of docosahexaenoic acid, a PUFA, compared to the non-infective procyclic forms (113). Docosahexaenoic acid and its derivative resolvin D1 were shown to induce M2 polarization in macrophages (117). In this context, it is interesting to note high serum levels of resolvin D1 and other lipids, such as prostaglandin F2 and leukotriene B4, observed in VL patients (118).

Activation of pro-inflammatory macrophages (M1) is due to stimuli such as IFN-γ, lipopolysaccharide (LPS), and TLR signaling (111, 119). Metabolomic studies, however, identified such activation to be dependent on upregulated glycolysis and glutaminolysis. Conversely, activation of anti-inflammatory macrophages (M2) occurs through stimuli such as IL-13 and IL-4, independently of TLR. Anti-inflammatory macrophage-activation is also dependent on fatty acid oxidation (**Figure 3**) (13, 120). Additional metabolic changes have been identified that drive subsequent immunomodulation of macrophages. For example, the upregulation of the pentose phosphate pathway, crucial for pro-inflammatory (M1) effector functions (121), occurs several hours prior to the induction of pro-inflammatory cytokines (122).

In pro-inflammatory (M1) macrophages, HIF-1α has been identified as the main regulator of glycolytic metabolism, and it contributes to pathogen clearance (**Figure 3**) (13). M1 macrophages upregulate anaerobic or aerobic glycolysis and the pentose phosphate pathway to generate ATP and NADPH, used for ROS production, which aids parasite clearance. On the other hand, M2 macrophages shift towards a more efficient mitochondrial respiration, making the macrophage more permissive to amastigote growth (14, 123). Additionally, arginine transport is upregulated in activated M1 macrophages, where it is readily used to produce NO. On the other hand, low IFN-γ activation leads to decrease NO levels and increased arginine availability for the parasite (14, 124). Taken together, these studies show that metabolic reprogramming of the host cell has a profound effect on its effector functions of macrophages.

Pro-inflammatory (M1) macrophages upregulate IL-12 and TNF-α expression, as well as ROS and RNS production, crucial to control *Leishmania* infection, as demonstrated in both mice and human models (111, 125, 126). Within this inflammatory microenvironment, IL-12 can upregulate iNOS and promote the development of IFN-γ-producing Th1 cells (99). IFN-γ then induces IL-12 and iNOS expression (99), which synthesizes high amounts of NO with leishmanicidal activities. However, the signal transduction of both IL-12 and IFN-γ in macrophages is interrupted in *Leishmania* infections (*L. donovani, L. major*, and *L. mexicana)* by inhibition of STAT-1 and STAT-4 phosphorylation (127, 128). Also, *Leishmania* favors the activation of SHP-1, an inhibitor of the TLR cascade, which impairs production of pro-inflammatory cytokines (**Figure 3**) (129). The importance of SHIP-1 pathway in parasite survival was demonstrated in invitro studies by the inhibition of SHIP-1 pathway by using antileishmanial drug sodium stibogluconate (130). Similarly, surface molecules such as lipophosphoglycan (LPG) from *L. donovani* dampen the IL-12 and ROS production by preventing MAPK activation (127), although the protective effect of LPG seems to be species and strain dependent (131, 132). A more in-depth discussion of the signaling pathways involved in *Leishmania* infection has been covered in detail in other reviews (74, 80, 82, 99).

Anti-inflammatory (M2) macrophages show an IL-10 and IL-4 dominant response that can lead to decrease NO secretion (7, 109, 133). Furthermore, IL-10 can make macrophages refractory to IFN-γ-mediated activation (99). In human VL, high IL-10 levels in blood and lesion tissue correlated with high parasitic load (134–139). Thus, human *L. donovani* infections show a predominant activity of M2 macrophages (140, 141) however M1/M2 dichotomy in protection *versus* pathogenicity is not always as binary. M2 macrophages can also play a protective role during VL, as IL-4 and IL-13 are necessary for collagen deposition and granuloma formation (**Figure 3**) (142).

### Role of Macrophages in Vaccine Induced Immunity

Due to the specialized role of macrophages in the induction of immune response against leishmaniasis, they are extensively studied in the context of assessing candidate vaccines. Macrophages due to their role of professional antigen presenting cells, are crucial for T cell activation after antigen presentation. The combined signals provided by the parasitized macrophages displaying *Leishmania* antigenic epitopes and their interaction with naïve CD4^+^ T cells bearing cognate T cell receptors lead to the development of an antigen-specific effector T-cell response. Accordingly, the role of macrophages in *Leishmania* clearance during challenge infection following activation of T effector cells has been reported in numerous experimental vaccines including protein-based, nano-particulate encapsulated, and DNA-based vaccines (143–147). The macrophage chemokine network is critical in producing tissue resident CD4+T memory cells (TRM) in viral vaccines (148). A similar role for macrophages in the generation of tissue resident memory T cells in *Leishmania* remains to be demonstrated.

The role of macrophages in the design and development of novel candidate vaccines has been reviewed previously (149, 150). Still, it is possible that vaccines or adjuvants targeting macrophages potentiate and complement the cellular immune response, at the same time allowing the macrophages to intensify the killing of internalized *Leishmania.* M1 macrophage-mediated induction of a polarized Th1 response was associated with resistance to intracellular pathogens. Such a polarization of T cells by pro-inflammatory (M1) macrophages was associated with protection in several vaccine studies, including studies of recombinant Mycobacterium bovis BCG, attenuated West Nile virus (WNV), and live attenuated measles virus. These studies show that efficacious candidate vaccines manipulate macrophages to enhance pro-inflammatory responses and yield improved protection (151–153). Exploitation of differential macrophage phenotype towards improving the efficacy of experimental vaccines warrants further studies.

The role of macrophages in potentiating a Th1 or Th2 response in vaccine immunity or pathogenesis has been shown in experimental *L. donovani* infections, with a special emphasis on the role of membrane cholesterol in enabling anti-leishmanial activities of macrophages (154). Specifically, *L. donovani* can alter the physiology of the macrophage membrane by depleting cholesterol, resulting in defective antigen presentation and impaired T cell responses (155–157). Interestingly, as opposed to virulent wild type *L. donovani* (*LdWT*) infection, immunization with the avirulent genetically modified *LdCen* ^-/-^ does not interfere with membrane fluidity and antigen presentation, allowing for macrophage activation and induction of Th1 immunity (120). Similarly, human macrophages infected with *LdWT* or *LdCen* ^-/-^ have shown differential expression of miR-21 and its target gene IL-12 illustrating the early immune modulatory role of the live attenuated vaccines (158). In summary, although macrophages have been studied extensively in VL pathogenesis and in experimental vaccines, emerging data in other vaccines exploring their role in trained immunity suggests that early metabolomic reprogramming in macrophages similarly could have profound effects in determining efficacy or *Leishmania* vaccines and therapeutics thus remain to be investigated.

## Dendritic Cells

**Figure 4:**
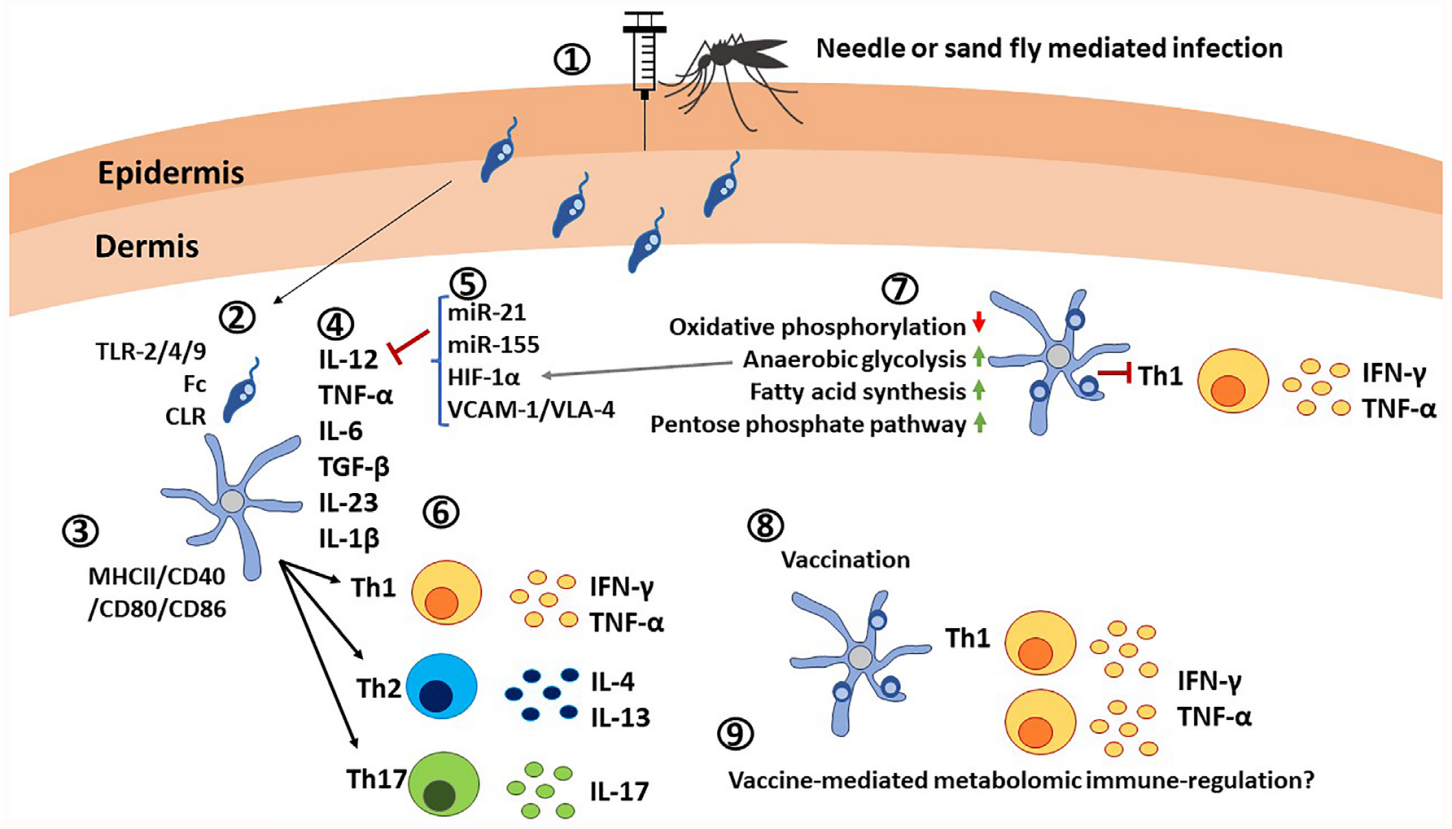
Critical roles of dendritic cells in immunity against *Leishmania* parasites. 1) *Leishmania* promastigotes are injected into the host *via* needle injection or sand fly injection. 2) DCs interact with *Leishmania* parasites through TLR-2/4/9, Fc receptors and C-type lectin receptors. 3) Activation of DCs is indicated by the elevated expression of MHC-II/CD40/CD80/CD86. *Leishmania* infections also causes expression of co-inhibitory molecules. 4) DCs are the main producers of IL-12 in addition to TNF-α, IL-6, TGF-β, IL-23, and IL-1β, which determine the differentiation of naïve T cells. 5) Expression of IL-12 is affected by microRNAs, transcription factors such as HIF-1α and ligands such as VCAM-1/VLA-4. 6) Upon interaction with DCs in lymphoid tissues in presence of cytokine signals, naive T cells differentiate into Th1, Th2 or Th17 cells and produce pro-inflammatory cytokines IFN-γ, TNF-α, or anti-inflammatory cytokines IL-4, IL-13 or IL-17. 7) Infected DCs are characterized by upregulation of anerobic glycolysis, fatty acid synthesis, pentose phosphate pathway, and by down regulation of oxidative phosphorylation, which results in poor expansion of Th1 cells. 8) Vaccination with live attenuated *Leishmania* parasites has shown elevated IL-12 production and expansion of Th1 cells. 9) Similar to other innate immune cells, metabolomic immune regulation in DCs upon vaccination remains an unexplored area.

Dendritic cells (DCs) are professional antigen-presenting cells (APCs), are ubiquitous in the peripheral tissues, and perform sentinel functions (**Figure 4**). Several distinct subsets of DCs (plasmacytoid, conventional, monocyte derived and more) have been identified based on their functional specialization and expression of distinct markers (159). Similar to other phagocytic cells, DCs can take up antigens *via* Fc receptors, C-type lectin receptors (CLRs), and pattern recognition receptors such as TLRs (**Figure 4**) (160). Recognition of *Leishmania* parasites by DCs is accomplished *via* TLR-2, -4, and -9 (161). TLR-9 has been identified as being responsible for DC activation and production of neutrophil chemo-attractants and IL-12 secretion in *L. infantum* infection in C57BL/6 mice (162). Following infection, DCs undergo maturation and express MHCII, CD80, CD86, CD40 and migrate to lymphoid tissues where they present antigens to naïve T cells (**Figure 4**) (10). DCs are the main producers of IL-12, which is critical for polarization of naïve T cells into IFN-γ producing Th1 cells (163). *L. donovani* infected DCs start producing IL-12, IL-23 and IL-27 within 5 hours of infection, IL-12 being mostly produced in CD8α^+^ DC subsets (164). In the chronic stage of *L. donovani* infection, CD11c^+^ splenic DCs showed a functional impairment, correlated with reduced surface expression of MHC-II and impaired IL-12 production (165). Similarly, migration of splenic DC into marginal zones is regulated by the CCR7 ligands CCL19/CCL21, which also affect the ability of DCs to produce IL-12 (166).

### Role of DCs in VL Pathogenesis

Expression of IL-12 in DCs is regulated by several factors including HIF-1α (167), interactions between VCAM-1/VLA-4 (Vascular cell adhesion molecule-1 and very late antigen-4) (168), miR-21 (169), and miR-155 (**Figure 4**) (170). DCs patrol for pathogens and participate in the immune response against *Leishmania* parasites, mainly by migrating to secondary lymphoid tissues and activating naïve T cells through antigen presentation. In addition, parasite factors such as *L. infantum* excreted/secreted proteins (LipESP) reduced the ability of human DCs to produce IL-12p70 (171). Similarly, exosomes from *L. donovani* failed to prime monocyte-derived human DCs to drive the differentiation of naive CD4 T cells into IFN-γ-producing Th1 cells *in vitro*. Vesicles from *L. donovani* parasites deficient in HSP100 showed a stronger pro-inflammatory phenotype in human DCs *in vitro* (172). Similarly, exosomes from *L. donovani*-infected bone marrow-derived DCs (BMDCs) showed a miR-21 dependent inhibition of IL-12 production indicating numerous mechanisms exist that regulate the function of DCs, particularly IL-12 production (158).

In addition to cytokine/chemokine-mediated immune regulation, metabolic changes in DCs have also been shown to affect their functions. For instance, inactivated DCs use oxidative phosphorylation and fatty acid oxidation for energy supply and biomolecule synthesis (173). However, infection leads to substantial metabolic changes as a result of PAMP-mediated activation. In particular, oxidative phosphorylation is reduced (174), while anaerobic glycolysis, fatty acid synthesis, and the pentose phosphate pathway are induced for ATP production and biosynthesis of lipids and nucleotides (**Figure 4**) (175, 176). These metabolic changes play important roles in inflammation and in the establishment of an immune response. Generally, the regulator of glycolytic metabolism HIF-1α plays a key role in DC maturation and activation during inflammation (177). However, HIF-1α was shown to promote *L. donovani* infection by reducing IL-12 production in splenic DCs, thereby limiting Th1 expansion (167), and impairing CD8+ expansion (178) in murine VL models. These findings are in contrast to what was shown in macrophages, where HIF-1α reduced susceptibility to *L. donovani* (179), and suggest that the effects of glycolysis are likely to be host cell specific.

### Role of DCs in Vaccine Induced Immunity

DCs due to their potent APC activities have been used as potential *Leishmania* vaccines by loading selected vaccine antigens or DC-derived exosomes, although these vaccines did not progress beyond laboratory studies (180–182). More recent studies focus on *ex-vivo* pulsing of DCs, and then using these cells to vaccinate mice, which has shown promising results. As such, pulsing the DCs with the N-terminal of *Leishmania* elongation factor 2, an antigen overlapping MHC-I and MHC-II epitopes, triggered a T cell response when used with CpG oligodeoxynucleotides. Such vaccinated mice could control *L. infantum* infection, resulting in IL-2 and TNF-α, but not IL-10 production by CD4+ T cells, and in the production of IFN-γ by both CD4+ and CD8+ T cells (183). DCs have also been shown to play a critical role in priming Th17 response in *L. donovani* infections. Immunization with *LdCen^-/-^* parasites showed that *LdCen^-/–^* infected DCs produce IL-1β, IL-6, and TGF-β to promote the development of Th17 lineage, and upon virulent challenge with wild-type *L. donovani* (LdWT) infection both CD4 and CD8 T cells produced IL-17, resulting in protection (184). Similar results were found when priming DCs with the C-terminal domain of nucleoside hydrolase NH36 from *L. donovani* (185). In addition to the T cell response against the *L. infantum chagasi* challenge, the defect in DCs migration to the lymph node was reversed, allowing proper antigen presentation (185).

The multifarious roles played by DCs as a critical source of IL-12 and the redundant mechanisms that affect the expression of IL-12 in *Leishmania* infection highlight the pivotal role of DCs in pathogenesis and potentiating an efficacious vaccine response.

## Mast Cells

Mast cells (MCs) have been shown to participate in the innate immune responses in CL and VL (8, 186). MCs participate in the initiation and orchestration of innate and adaptive defense to pathogens and in various inflammatory responses (187, 188). MCs are present in large numbers in the skin, predominantly in the superficial dermis, the site where *Leishmania* parasites are deposited after the bite of infected sand flies (189, 190).

Literature on MCs with respect to visceral infections of *Leishmania* is sparse. Much of our understanding of the role of MCs in *Leishmania* is derived from studies of CL. MC-derived TNF-α followed by neutrophil influx and MIP-1α/β release is required for the recruitment of macrophages during cutaneous granuloma formation, a hallmark of parasite-induced inflammatory responses (191). Indeed, MCs recruit effector cells of innate and adaptive immunity to sites of *L. major* infection and induce systemic protective dendritic cell (DC)-dependent adaptive immune responses that eventually control *L. major* infection. Differential MC infiltration patterns and responses have been reported depending on the mouse strains (8) and *Leishmania* species (186). For example, there was a significant uptake and killing of *L. tropica* by MCs compared to *L. donovani*. Interactions of MCs with both these *Leishmania* species ensues the release of MC extracellular traps (MCETs) analogous to NETs produced by neutrophils (**Figure 1**). Although both *L. donovani* and *L. tropica* seem susceptible to MCETs, relatively higher number of viable promastigotes of *L. donovani* in comparison to those of *L. tropica* are observed indicating the relative resistance of *L. donovani* parasites. Thus, MCs play a very important role in early innate immune response to *L. tropica* and *L. donovani* and thus may play an important role in the success of vaccines against Leishmaniasis (186). Indeed, it has been hypothesized that MC-induced control of *L. major* infections is not only restricted to the induction of local inflammation, but that MC recruitment of pro-inflammatory cells (*i.e.* DCs) to sites of *L. major* inoculation ensures the development of protective, long-lasting memory responses against *L. major* (192), suggesting the relevance of MCs in vaccine immunity. Interestingly, vaccination with *LdCen* ^-/-^ parasites against virulent *L. mexicana* challenge resulted in reduced infiltration or absence of degranulated MCs, which correlated with protection (193). MCs may have a role in *Leishmania* vaccination, similar to that observed in BCG vaccination, where MCs phagocytize BCG, produce ROS, and release MCETs (194). More studies in experimental models and in humans are required to understand the immune mechanisms that determine MCs involvement in *Leishmania* vaccine-induced immunity. MCs-derived molecules have shown potent adjuvant activity in certain vaccines [Reviewed in (195, 196)]. In *Toxoplasma gondii* infection, the treatment with two different MCs-derived molecules, C48/80 or cromoglycate, can either increase or decrease the parasite burden by differentially favoring Th1 or Th2 responses, respectively (197). Similar results have also been observed in a *Trypanosoma cruzi* murine model, where cromoglycate administration led to an increase of parasitic burden (198). Administration of other MCs-derived molecules like tryptase, resulted in the cleavage of many Th2 cytokines, and consequent Th1 polarization (199). Similar studies in *Leishmania* exploring the role of MC-derived molecules as potential immunomodulators remain to be undertaken.

## Natural Killer and NKT Cells

Natural killer (NK) cells, identified as CD3−CD56+ in humans, are characterized by their cytotoxic activity and cytokine production (200), and have been shown to participate in the immune response against *Leishmania* (**Figure 1**). VL patients show three different NK subsets: CD56–CD161+, CD56+CD161–, and CD56+CD161+, with a loss of the CD56+CD161+ population in comparison to the healthy individuals (201). TLR-2 recognition of *Leishmania* LPG activates NK cells and induces the production of IFN-γ and TNF-α and the translocation of NF-κB to the nucleus. This activation seems to be more intense in response to metacyclic promastigotes, compared to procyclic promastigotes (202). IFN-γ production and NK cytotoxicity is also dependent on TLR-9 (203). Interestingly, the downregulation of STAT-1 related to the reduction in IFN-γ, TNF-α and TLR2 expression by NK cells allows the development of diffuse cutaneous leishmaniasis (DCL), a form of the disease characterized by the uncontrolled spread of the parasites (204). Moreover, lesion healing of DCL patients corelates with the increase of CD16+ CD56+ NK cells (205). Despite their important role, NK cells are not necessary for the establishment of an effective Th1 response against *L. major* (206), or *L. tropica*, whereas they are indispensable for the elimination of *L. donovani* amastigotes (207). A more detailed appraisal of the role of NK cells during leishmaniasis can be found in (208). Recent studies have identified NK cells to play a role in vaccine-mediated immunity. Laabs, et al. have shown that vaccination with live *L. major* combined with CpG DNA leads to DC and NK activation, as well as increased IFN-γ production by NK cells (209). Interestingly, NK cells have also been shown to acquire trained immunity, in the context of BCG vaccination. However, the exact epigenetic and metabolic reprogramming underlying this phenomenon remains to be fully elucidated (210, 211). Similar to other innate cell types, the role of trained immunity in NK cells needs to be further investigated in the context of *Leishmania* infection and vaccination.

There are few reports about the role of NK-T cells (NKTs) in leishmaniasis. NKTs are CD1d-restricted T cells with innate and adaptive properties, which react to a variety of stress proteins and glycolipids using either NK or T cell effector mechanisms (212). Glycoconjugates on the *Leishmania* surface are detected by CD1d+ NKT cells, which are protective against leishmaniasis in early stages of VL (213). CD8+ NKT cells are also protective, express IFN-γ and Killer cell immunoglobulin-like receptors (KIRs) and do not migrate towards the *L. donovani* infection site, whereas the CD4+ NKTs are found to be pathogenic as they migrate towards the infection site and express CD25, FoxP3 and IL-10 (214). In comparison to other immune cells, literature on the role of NK and NKT cells remains limited in VL. They may yet play important roles in protective immunity in anti-leishmania vaccines analogous to the limited studies in BCG vaccination that revealed potent trained immunity that contributed to protection.

## Conclusion

The immune response to *Leishmania* infection is primarily initiated by innate immune cells which orchestrate the generation of protective innate and adaptive immunity against *Leishmania* parasites. Innate immune cells specialize in the clearance of invading pathogens. *Leishmania* parasites have evolved a range of evasion strategies to subvert normal innate cell function. While the role of the innate immune cells in host protection during *Leishmania* infection are being explored, important questions regarding their roles in shaping the protective immunity in a prophylactic vaccine setting remain to be investigated. Furthermore, chronic infections with *Leishmania* parasites have been shown to induce prominent changes in host metabolomic pathways that influence immune cell proliferation, differentiation, and effector functions. Current advances in metabolomics have made significant impact and present important implications in the management of VL. Metabolomic profiling during VL may reveal novel immune regulation networks that can be exploited for vaccine development. Additionally, while metabolic changes in different *Leishmania* species are well studied and could serve as biomarkers of virulence and disease progression, what would be a desirable metabolic profile for innate immune cells during vaccination remains to be investigated. In conclusion, innate immune cells are critical for the development protective immunity due to their profound role in shaping adaptive immunity. Furthermore, innate cells themselves may undergo epigenetic and metabolic changes that renders them to acquire trained immunity, an emerging concept that has been studied in various vaccines. Such studies of metabolomic and epigenetic changes of innate immune cells will benefit the development of efficacious vaccines against VL.

## Author Contributions

GV, TPF, PB, TO, and SG drafted the article. ARS and HLN conceived the theme of the article. HLN and SG reviewed the draft. All authors contributed to the article and approved the submitted version.

## Funding

This work is supported by NIH/NIAID grants R21 AI130485 02, RO3 AI144253 01 (ARS), Global Health Innovative Technology Fund grants G2018-201 and G2019-213 (ARS, HLN) and by intramural funding from FDA.

## Author Disclaimer

Contributions by the FDA authors are an informal communication and represent their best judgment. These comments do not bind or obligate FDA.

## Conflict of Interest

The authors declare that the research was conducted in the absence of any commercial or financial relationships that could be construed as a potential conflict of interest.

## Publisher’s Note

All claims expressed in this article are solely those of the authors and do not necessarily represent those of their affiliated organizations, or those of the publisher, the editors and the reviewers. Any product that may be evaluated in this article, or claim that may be made by its manufacturer, is not guaranteed or endorsed by the publisher.
